# Personalized Joint Replacement: Landmark-Free Morphometric Analysis of Distal Radii

**DOI:** 10.3390/jfmk10010071

**Published:** 2025-02-21

**Authors:** Sarah L. Remus, Kevin Brugetti, Veronika A. Zimmer, Nina Hesse, Paul L. Reidler, Riccardo Giunta, Julia A. Schnabel, Wolfram Demmer

**Affiliations:** 1 School of Computation, Information and Technology, Technical University of Munich, 80333 Munich, Germany; 2 Department of Hand, Plastic and Aesthetic Surgery, LMU Klinikum, Ziemssenstraße 5, 80336 Munich, Germany; 3 School of Medicine and Health, Technical University of Munich, 80333 Munich, Germany; 4 Department of Radiology, LMU University Hospital, LMU Munich, 81377 Munich, Germany; 5 Institute of Machine Learning in Biomedical Imaging, Helmholtz Munich, 85764 Neuherberg, Germany

**Keywords:** anatomy of the wrist, landmark-free morphometry, CT, 3D-printed prostheses, distal radius, mirror symmetry, hand surgery

## Abstract

**Background:** Fractures of the distal radius are common, particularly among young men and elderly women, often leading to painful wrist arthritis, especially if the joint surface has been affected. Traditional treatments of the wrist, such as full or partial wrist fusion, limit movement, and common wrist prostheses have high complication rates. Regenerative medicine and 3D bioprinting offer the potential for personalized joint replacements. **Methods**: This study evaluates using the contralateral radius as a template for creating customized distal radius prostheses. Bilateral CT scans of healthy wrists were analyzed to assess the shape and symmetry of the distal radius using a landmark-free morphometric method. Instead of comparing defined landmarks, the entire surface of the radius is analyzed employing dense point- and deformation-based morphometry to detect subtle morphological differences, providing an unbiased and more accurate comparison of the overall deformations in the distal radii. **Results:** results show strong intraindividual symmetry in joint surfaces. Interindividual comparisons revealed significant morphological variations, particularly gender-specific differences. **Conclusions:** These findings support the use of the contralateral radius as a template for the replaced side. At the same time, the interindividual results endorse the approach of pursuing personalized prostheses as the optimal replacement for distal joint surfaces. The increasing improvement of 3D-printed prostheses promises new methods for better outcomes in distal radius arthrosis after intraarticular fractures. Further research into clinical applications and biocompatible 3D printing materials is recommended.

## 1. Introduction

Fractures of the distal radius are the most common fractures in the Western world [1]. In about one-third of cases, the joint surface of the distal radius is affected, with partial or complete joint involvement (23-B and 23-C fractures, AO-Classification) [1]. Many distal radius fractures heal insufficiently [2], often leading to painful and limiting wrist arthritis [3]. Treatment for this subsequent damage typically consists of partial or complete wrist fusion, leading to significant restriction in movement [3]. Wrist prostheses, while preserving some mobility, have not become standard due to high complication rates, including issues like implant loosening and bone erosion [4,5,6,7,8,9,10].

In cases of complex distal radius fractures accompanied by joint damage, reconstructive procedures often necessitate a reference point to reconstruct the original joint configuration. Typically, surgeons rely on the contralateral radius as a reference, presuming it closely mirrors the injured radius. Several studies have explored the symmetry between the left and right radii. For instance, Vroemen et al. [11] and Dobbe et al. [12] investigated the overall symmetry within individuals, revealing notable variations in length and rotation.

Advances in the field of regenerative medicine and 3D bioprinting offer promising approaches to bring the perspective of individualized bio-integrative bone cartilage substitutes for joint replacement within reach [13]. Using the contralateral radius of the patient as an anatomical template is a plausible approach [14]. A prosthesis of the distal surface could then be designed using a CT scan of the patient’s unaffected wrist and produced by 3D printing technology.

Several studies have shown that small misalignments of fracture fragments greater than 1–2 mm, particularly when involving the articular surface, are linked to significantly poorer clinical outcomes, such as osteoarthritis or limited range of motion. Ensuring the accurate anatomical reconstruction of the joint is crucial and highly correlated with better postoperative results [2,15,16,17]. In our analysis, we, therefore, established a cut-off value of 1 mm, below which we can expect very good clinical outcomes [2].

Mirror symmetry in a patient’s body is generally assumed. However, in the medical literature, the mirror symmetry of the distal radius is discussed controversially [11,18]. Most studies focus on the larger metaphysis of the radius rather than on the distal radius console and the joint configuration. Gray et al. conducted a shape analysis of the distal radius by identifying seven anatomical landmarks, finding no statistically significant differences measured between the right and left distal radius in 37 paired wrists [19]. This landmark-based shape analysis approach has limitations in capturing the complete shape of the distal radius and especially the joint surface. We, therefore, employ a landmark-free method, offering a promising solution by providing automated, high-resolution analyses without the need for manually placed landmarks. The presented method improves spatial resolution and the fidelity of shape-difference visualization and mitigates the need to separate size and shape changes, ultimately enhancing the precision and efficiency of shape analysis compared to manual landmark-based approaches, allowing for a fully automated comparison of segmented distal radii [20]. This method was already successfully used to study mandibular symphyseal shapes [21] and craniofacial skeletons of mice [20].

In this study, we focus on the mirror symmetry of the bilateral radii within the same individual as well as the expression of interindividual differences in the cohort using a landmark-free morphometrical analysis for shape comparison. This is done by conducting an intra-individual analysis, referred to as the “pairwise method”, as well as an interindividual analysis, referred to as the “atlas method”. The information obtained allows us to conclude whether the contralateral radius can serve as a template for 3D-printed prostheses and inform whether personalization of distal radius joint prosthesis is recommendable due to interindividual differences.

## 2. Materials and Methods

The data for this study was provided by the hospital of the Ludwig-Maximilians-University Munich (LMU), adhering to the principles outlined in the “Declaration of Helsinki” and receiving approval from the Ethics Committee of LMU (Project No. 22-0674 (Date 26 August 2022)).

### 2.1. Study Population and Acquisition of Imaging Data

Data collection involved trauma spiral computed tomography (CT) scans of patients treated in the trauma room between 2012 and 2022. Since, in most cases, wrists were not injured, a trauma spiral CT was an ideal way to obtain bilateral scans of patients’ wrists.

The study included patients aged 18 to 80 years with undamaged distal radius articular surfaces in age-appropriate normal conditions. Eligible patients had undergone trauma spiral CT scans between 2012 and 2022 as part of emergency care, with wrists clearly visible and adequately resolved. To obtain a random and thus unbiased selection of CT scans, they were screened in chronological reverse order (latest scans first) for adequate evaluation regarding image quality. Only afterward were the patients’ gender and age recorded. To create comparable cohorts, random records of men and women, as well as individuals from the age groups 20–40 and 40–80 years, were selected to form equal-sized groups. This approach allowed for the identification of both potential gender-specific and age-specific differences. Exclusion criteria were patients whose CT scans were unsuitable for evaluation due to technical reasons, such as insufficient resolution, artifacts, or incomplete depiction of the wrists. Additionally, patients with fractures of the distal wrist or other severe damage to the distal radius articular surface were excluded from the study. No further inclusion or exclusion criteria were applied.

A SIEMENS “Somatom Force scanner” (Siemens Healthcare GmbH, Forchheim, Germany) was used for CT scans. For consistent image quality, the venous phase was chosen as default. Each CT scan possesses a pixel spacing of 0.869/0.868, signifying that horizontally adjacent pixels are approximately 0.869 mm apart, while vertically adjacent pixels are about 0.868 mm apart. A window thickness of 0.75 mm was set for the CT scans.

**Figure 1:**
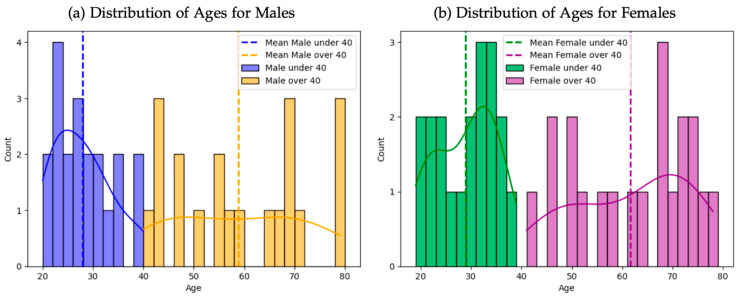
Age distribution of male (**a**) and female (**b**) patients.

Out of all the CT scans, a total of 4 cohorts were created, each consisting of 10 patients. The cohorts were matched by gender and age to facilitate specific intra-individual comparison: 10 male patients aged 20–40, 10 female patients aged 20–40, 10 male patients aged 40–80, and 10 female patients aged 40–80 (Figure 1). The age threshold of 40 was chosen as it marks the presumed onset of idiopathic joint degeneration [22]. The data was anonymized and exported in DICOM format.

### 2.2. Mesh Extraction

“Medical Imaging Interaction Toolkit” (MITK Workbench, German Cancer Research Center, Heidelberg, Germany, v2021.10, MacOS) software was used to crop the images and isolate the distal radius, extracting the region of interest from the full-body scans. To standardize the images, the radii underwent coronal plane reconstruction using the multiplanar reformation (MPR) tool within MITK. Following this, manual segmentation on all 80 radii was performed by a medical expert. Each layer was segmented separately and without the use of automated segmentation assistance [23]. Using these segmentation labels, 3D surface meshes of the distal radii were generated for each patient. These meshes were created by using the “Marching Cube Algorithm” utilizing the “Visualization Toolkit” (VTK, Kitware Inc., New York, NY, USA, v.8.2.0, MacOS) and “Python”. We define the shapes as polygonal meshes with vertices, edges, and faces. Each vertex is connected to at least one edge, each edge to at least one face, and any intersection of faces must occur at a shared vertex or edge, ensuring no isolated vertices, edges, or face interpenetration [24].

### 2.3. Data Preprocessing

**Figure 2:**
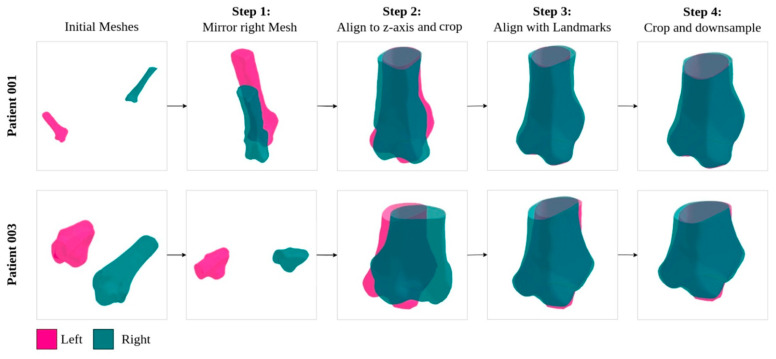
Pairwise preprocessing of distal radius segmentation meshes. Step 1: Mirror right mesh over the *x*-axis. Step 2: Align meshes along the *z*-axis using Rodrigues’ rotation formula, move the lowest point in the mesh to the (0, 0, 0) position and crop meshes at 5 cm. Step 3: Align left and right radii by aligning the two landmarks RS and DSN. Step 4: Crop meshes to 4.3 cm and down sample meshes to around 5295 ± (SD: 30) number of points.

To adequately capture shape variation among the different radii, it is essential to align the shapes to exclude position and orientation from the analysis. Global alignment is crucial in shape analysis to ensure that variations in position, orientation, or scale do not interfere with the accurate comparison of anatomical structures. By aligning the distal radius meshes, we can focus on shape differences rather than discrepancies caused by differing positions or orientations during imaging. The preprocessing for the distal radius shape analysis consists of four key operations (Figure 2), aiming to standardize the meshes and enable a more precise and meaningful analysis of the anatomical features under study.

First, the meshes of the right wrist were subjected to a mirroring operation over the *x*-axis, effectively transforming it into its mirror image. The mesh was mirrored across the *x*-axis using a transformation matrix, which negated the x-coordinates while keeping the y and z coordinates intact. This resulted in a mirrored mesh, and the same transformation was applied to the corresponding landmarks to preserve their spatial relationships. This ensured consistency between the mesh and its landmarks during the transformation. The decision to deform the right mesh into the left was arbitrary.

To rectify initial misalignment in 3D space caused by the random position of both radii during the CT scan, the meshes were aligned along the *z*-axis using “Rodrigues’ rotation formula” [25]. Employing principal component analysis (PCA) enables this alignment by projecting the data onto a principal subspace, preserving maximal variance [26]. Subsequently, a rotation matrix was derived to align the relevant axes. Applying this rotation matrix to both the surface mesh and landmark points ensures consistent alignment. The mesh was aligned along the *z*-axis by shifting the lowest point of each mesh to the (0, 0, 0) coordinate. Both meshes were then cropped at the 5 cm mark using a cutting plane at (0, 0, 50) with a normal vector (0, 0, 1). This ensured consistent mesh length (5 cm), and the same adjustment was applied to the corresponding landmarks for consistency in the 3D space.

**Figure 3:**
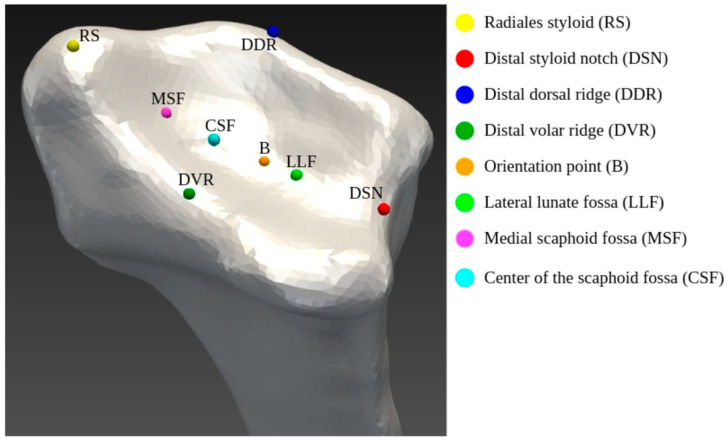
Anatomical landmarks including abbreviations and their positions on an exemplary articular surface of the distal radius. For alignment, only Radial styloid (RS) and distal styloid notch (DSN) were used.

Anatomical landmarks on the articular surface of the distal radius were used to achieve a robust, rigid alignment. Gray et al. described 8 landmarks to define the distal radial articular surface [19]. In our study, we used two of those landmarks, the Radial styloid (RS) and distal styloid notch (DSN), to align the meshes of the distal radii (Figure 3). To optimize alignment between the anatomical landmarks of the RS and DSN, situated on opposite sides of the articular surface, we additionally calculated the centroids of each mesh and aligned the shapes along these 3 reference points. RS and DSN are located at anatomically well-defined positions, making them a reliable solution for alignment. The landmark data for each patient was initially stored in MPS files and subsequently converted into VTK point cloud data. Furthermore the “least squares problem” was solved to determine the linear transformation for the best fit between these points to counteract potential rotation discrepancies.

After aligning landmarks and centroids, meshes were cropped to ensure consistent termination points. This way, variations in termination areas did not affect subsequent shape analysis. Meshes were resized to 4.3 cm for uniformity and down-sampled to ensure comparability. Post-reduction, all 80 meshes contained an average of 5295 ± 30 vertices.

Furthermore, the articular surface was isolated from the distal radius for a focused statistical analysis. A defined plane was drawn perpendicular to the *z*-axis and aligned with the DSN landmark to define and isolate the articular surface [19].

Additionally, for the atlas shape analysis, each mesh was aligned with the right distal radius mesh of the first patient using the RS, DSN, DDR (Figure 3), and the centroid of each mesh. To standardize the meshes, they were all cropped to a uniform length of 4.3 cm, ensuring the inclusion of the region of interest (distal radial epiphysis) and preventing the interference of proximal parts of the radius. The polygonal mesh was loaded and converted into a PolyData object using PyVista (Kitware Inc., New York, NY, USA, v.0.42.3). The target reduction ratio was calculated, and PyVista’s decimate method was applied to efficiently reduce the mesh’s resolution while preserving its original shape. Post-reduction, all 80 meshes contained an average of 5295 ± 24 vertices.

### 2.4. Proposed Method

The landmark-free method for shape analysis applied in this study is based on a shape alignment method first introduced by Durrleman et al. in 2014 [27]. Two shapes are aligned using a flow of diffeomorphisms (smooth and invertible deformations). This allows finding the correspondences between two shapes by deforming one shape to the other using thus shape alignment method. The variations in shapes are captured in these deformations and can be used to analyze shape differences of the distal radii. In the following, we compare two different approaches, for pairwise and for atlas shape analysis.

#### 2.4.1. Pairwise Shape Analysis

##### Shape Alignment Using Diffeomorphisms

Once the right and left surface meshes of each patient were accurately aligned, the right distal radius mesh was deformed to match the left counterpart using “*Deformetrica*” (ARAMIS Lab, Paris, France, v4.0, Python API) software (See Table 1 for parameter settings. The source code can be found under ‘Parameter’ at: https://gitlab.com/icm-institute/aramislab/deformetrica (accessed on 1 February 2025)) [27]. This is achieved by representing mesh A as a deformation of mesh B. In this way, the initially non-corresponding meshes are brought into point-to-point correspondence, as mesh A is expressed as a deformed version of mesh B. Since mesh B and its deformed version inherently have point-to-point correspondence, this allows for direct shape comparison between the meshes. We employed a registration method, which is a specific instance of the deterministic atlas method [28].

##### Landmark-Free Morphometric Analysis

A landmark-free method in shape analysis refers to a technique that does not rely on predefined anatomical landmarks for comparing and analyzing shapes. Unlike traditional geometric morphometrics (GMM) or typical landmark-based methods, where specific points on the anatomy (landmarks) are used as reference points to capture shape information, landmark-free methods focus on the overall deformations required to map one shape onto another. This approach involves computing the deformations between entire shapes rather than analyzing the positions of selected landmarks. By using PCA to study these deformations, the method provides a comprehensive understanding of shape variation that captures subtle differences across the entire structure rather than being limited to specific predefined points. A principal component is a linear combination of the original variables in a dataset, constructed in such a way that it captures the maximum possible variance. In PCA, the first principal component accounts for the largest amount of variance, with each subsequent component capturing the remaining variance while being orthogonal to the previous components.

In our study, we used this landmark-free method to analyze the difference between the patient’s left and right distal radius. Specifically, we used kernel PCA (kPCA) to analyze the so-called momenta vectors, which provide comprehensive deformation descriptors. This non-linear dimensional reduction method is suitable for scenarios characterized by non-linear separability [26,29]. Before applying kPCA to our data set, we standardized the momenta vectors to ensure equal contribution from all features. The radial basis function (RBF) kernel was used for the kPCA. We assessed the concordance between the left and right distal radius across patient cohorts.

To evaluate the normality of the data distribution for each PC, the Shapiro–Wilk test was performed. This test determines whether the data points of each PC adhere to a normal distribution, aiding in selecting appropriate statistical tests. The rejection of the null hypothesis for the first PC indicates deviations from normal distribution. Graphical assessments, such as Q-Q plots, further support the non-normality of the data. Considering the non-normal distribution of the data, we employed the Kruskal–Wallis test, a non-parametric statistical method, to examine whether the PC effectively captured meaningful variance across age and gender groups. A significance level of 0.05 was set for the test.

#### 2.4.2. Atlas Shape Analysis

As an alternative to pairwise shape analysis, we propose to use an atlas shape analysis, where an atlas may be regarded as the mean shape of a given set of shape complexes. In the atlas model, the mean shape for a group of objects and the deformation mapping of the atlas to each subject are estimated. The atlas is a key component of the interindividual comparison of the distal radius.

##### Shape Alignment Using Diffeomorphisms

To explore the variation in distal radius shape across the population, a shape analysis was conducted comparing a mean shape, termed the atlas, against individual patient meshes by using the deterministic atlas method provided by *Deformetrica*.

To construct the atlas shape to achieve point-to-point correspondence across all meshes, the deterministic atlas method was applied to every mesh while using the mesh from patient 1 as a template. After deformation, we computed the mean position for each point across all meshes, merging them into a new mesh. This new mesh became the initial template for our atlas shape analysis. Additionally, landmarks from all patients were used to calculate the mean landmark points for the distal radius to define the mean articular surface.

##### Landmark-Free Morphometric Analysis

We assessed shape variations across the population by calculating the signed Euclidean distance between the mean distal radius mesh and each patient’s distal radius mesh. Additionally, we used kPCA on the momenta vectors to identify differences among the four age and gender groups. The reliance on kPCA for dimensionality reduction assumes linear independence of features, which may not fully reflect complex biological shapes. In order to capture more of the underlying structure of the data and thus minimize concerns about loss of information or reduced accuracy in shape and symmetry comparisons, we, therefore, used a Gaussian kernel in our approach, designed to capture non-linear relationships between features of complex biological shapes. The Gaussian kernel maps the data into a higher-dimensional space, enabling kPCA to detect and preserve non-linear correlations between features that linear methods might miss. This approach minimizes the risk of overlooking important correlations in the morphological characteristics of the distal radius. Subsequently, we conduct Welch’s *t*-test to assess the statistical significance of these disparities.

## 3. Results

The experiments were carried out on a Linux workstation running Ubuntu (Canonical Ltd., London, UK, v.22.04). The workstation featured robust hardware, including 130 GiB of RAM and an AMD EPYC 7502P processor with 32 cores. An NVIDIA RTX A6000 graphics card was used with 32 GB RAM and CUDA (NVIDIA Corporation, Santa Clara, CA 9505 USA, v.12.1) to enhance our computational capabilities for accelerated processing. The above-mentioned Python API of Deformetrica version 4.0 was used to perform the shape analysis with Python (Python Software Foundation, Wilmington, DE, USA, v.3.8). For efficient manipulation of mesh data in Python, the Visualisation Toolkit (Kitware Inc., New York, NY, USA v.9.2.6) and its Python helper library, PyVista (Kitware Inc., New York, NY, USA, v.0.42.3) was used. These tools ensured seamless handling and processing of the complex shape data, allowing for in-depth analysis and precise results.

The settings of Deformetrica are shown in Table 1.

### 3.1. Pairwise Shape Analysis

Here, we present the results for the analysis of intraindividual mirror symmetry. For this pairwise shape analysis, the fact that the estimated left mesh corresponds point-to-point with the template right mesh was exploited. For simplicity, we will refer to the deformed right mesh as the “left mesh”.

Table 2a presents the mean Euclidean distance between the meshes along with their standard deviation. The mean distance between the left and right meshes for all patients is 0.53 ± 0.39 mm. Notably, males over 40 exhibit the highest mean distance between left and right (0.57 ± 0.43 mm), while females under 40 show the lowest (0.45 ± 0.34 mm).

**Figure 4:**
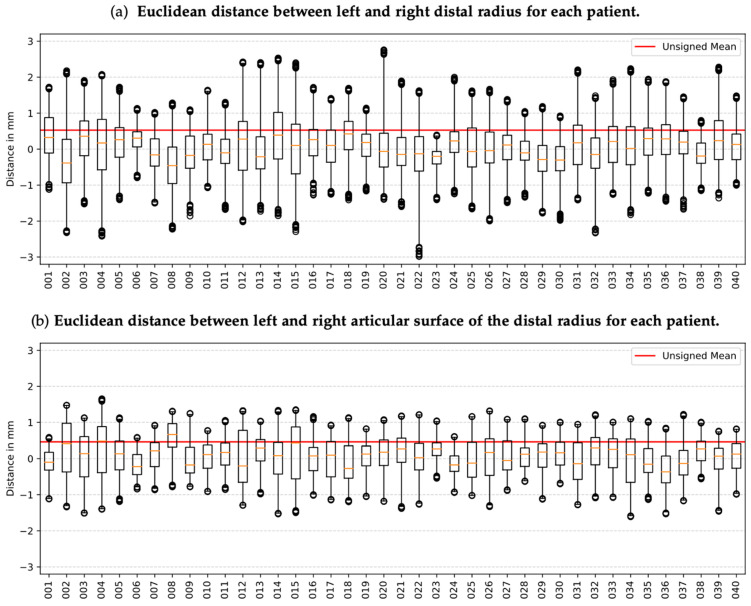
Pairwise signed Euclidean distance of (**a**) distal radius and (**b**) articular surface. Negative distances correspond to an inward deformation of the right mesh to match the left mesh, and positive distances indicate an outward deformation. The red line visualizes the mean distance between all pairs. The orange lines depict the median distance between the meshes of a patient. Outliers are the highest 99.8% distance values for each patient.

Figure 4a presents the signed Euclidean distance between corresponding points of the left and right distal radius. Negative values indicate required inward deformation of the right mesh to align with the left mesh, whereas positive values indicate outward deformation. Across patients, at least 50% of the distances between points of the left and right radius measure less than 1.00 mm. The most significant distance between two corresponding points in the left and right mesh is 3.00 mm between the radii of patient 22.

Figure 4b and Table 2b present analogous information for the isolated articular surface of the distal radius. The mean distance between left and right is 0.46 ± 0.30 mm, 0.07 mm less than the mean distance between the entire distal radii. Similar to the overall analysis, males over 40 exhibit the highest distance between left and right (0.50 ± 0.33 mm), while females under 40 display the lowest (0.40 ± 0.28 mm). Notably, all distances between corresponding points are less than 1.8 mm.

It is worth noting that despite the mean distance between the articular surfaces being only 0.07 mm less than the mean distance of the entire distal radii, the distances on the articular surface cover a much narrower range. In Group (a), 17.5% (7 out of 40) of patients exhibit distances <1.50 mm, while in Group (b), this percentage increases to 85% (34 out of 40).

**Figure 5:**
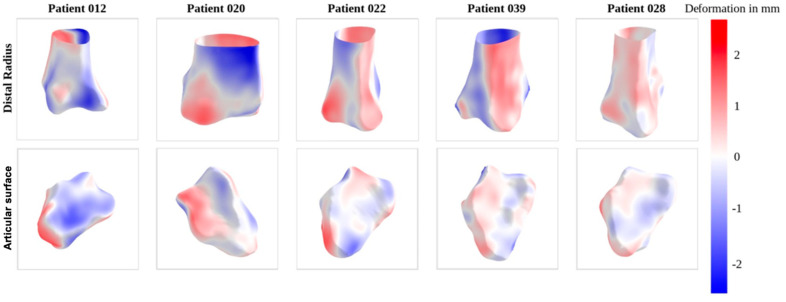
Example meshes for the pairwise shape analysis, illustrating the signed Euclidean distance between left and right distal radius. Front view of the whole radius as well as the articular surface. Meshes are sorted by mean Euclidean distance decreasing from left to right. Patient 12 has the highest mean Euclidean distance and patient 28 has the lowest. Red areas indicate an outward deformation, blue areas an inward deformation.

Figure 5 visually depicts the deformations observed in the radii of five selected patients. Notably, patient 12 demonstrates the highest mean distance, contrasting with patient 28, which exhibits the lowest mean distance. Patients 22 and 39 showcase notable outliers (>2.5 mm). Upon closer examination of the illustrations, it becomes apparent that the predominant deformations mostly occur within the metaphyseal and proximal epiphyseal regions rather than at the articular surface.

#### Analysis of Deformations

The differences among the four age and gender groups were analyzed using momenta vectors that describe the deformation from the distal radius. These vectors capture nuanced shape variances beyond mere depth alterations.

Results indicated that only the first PC exhibits a statistically significant difference among the age and gender groups (Table 3).

Post-hoc analyses were conducted using Welch’s *t*-tests to explore specific group differences. The first PC demonstrates significant differences between males under 40 and females under 40, males over 40 and females under 40, and males over 40 and females over 40, as evidenced by *p*-values below 0.05, suggesting notable distinctions in their means (Table 3).

### 3.2. Atlas Shape Analysis

**Figure 6:**
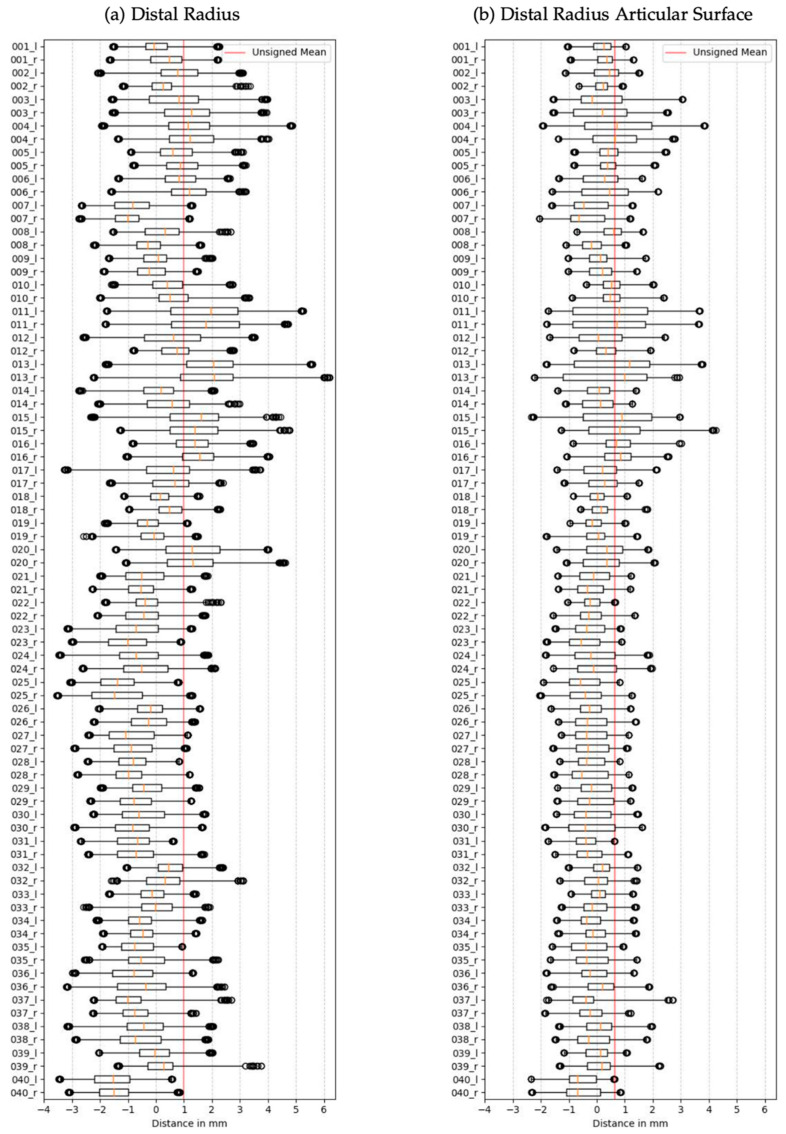
Signed Euclidean distance of (**a**) distal radius and (**b**) distal radius articular surface between patient distal radius and mean distal radius.

The mean Euclidean distance between all patient meshes and the atlas mesh comprised 0.98 ± 0.72 mm (Table 4 and Figure 6). Similar to the pairwise shape analysis, males over 40 exhibit the highest distance to the mean mesh among the four age and gender groups. The mean Euclidean distance between the articular surface meshes and the atlas shapes is 0.65 ± 0.49 mm. Unlike the pairwise shape analysis, some points still exhibit considerable distances between the atlas and patient articular surfaces, which suggests a higher variability in the articular surfaces within the population.

The observed deviation in the interindividual comparison of the articular surface (0.65 ± 0.49 mm) is 0.19 mm higher than the typical intra-individual difference between the left and right distal radii articular surfaces (0.46 ± 0.30 mm) in patients, where intra-individual differences remain consistently below 1 mm .

**Figure 7:**
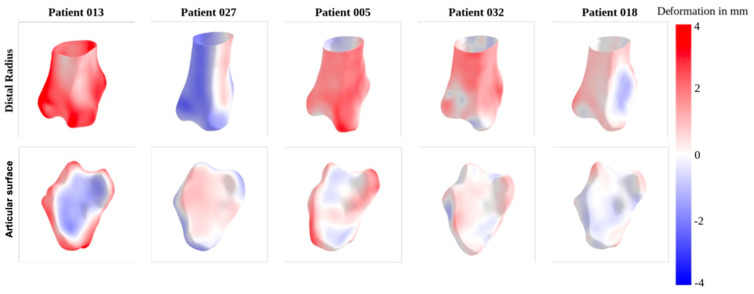
Example meshes for the atlas shape analysis illustrating the signed Euclidean distance between the atlas radius and left distal radius of a patient. The front view of the distal radius, the articular surface. Meshes are sorted by mean Euclidean distance decreasing from left to right. Patient 13 has the highest mean Euclidean distance between the left and the mean radius, and patient 18 has the lowest. Red areas indicate an outward deformation, blue areas an inward deformation.

Figure 7 illustrates the deformation patterns observed in five selected patients. Notably, patient 13 and patient 27 exhibit the most pronounced differences in size when compared to the atlas model. Patient 013 displays significant outward deformation in the metaphyseal region and substantial inward deformation on the articular surface. On the other hand, patient 027 shows a marked inward deformation in the metaphyseal region and a small outward deformation on the articular surface.

#### Momenta Vectors

Observing the eigenvalues of the PCs, it was found that the first PC captured significant variance in the data (Table 5). The Shapiro–Wilk test revealed that the PC data did not follow a normal distribution. Therefore, the Kruskal–Wallis test was used to examine whether the PCs effectively captured meaningful variance across age and gender groups. The first PC indeed captured significant variance, indicating their relevance in distinguishing between the groups.

Post-hoc analysis using Welch’s *t*-tests showed significant differences between most pairs of groups for the first principal component (Table 5). The *p*-values were notably small, underscoring the differences between the groups. A significance level of 0.05 was set for the test.

Visual examination revealed more distinct differences between groups compared to the pairwise analysis. Notably, there was a clear separation between females and males of both age groups. Welch’s *t*-tests confirmed significant differences in deformation between the mean distal radius and patient meshes for both genders.

## 4. Discussion

In order to archive a comprehensive morphometric analysis of the entire distal radius and its articular surface, rather than limiting the assessment to fixed measurements like rotation, length, or angles, we employed this procedure using landmark-free morphometrics. Landmark-based methods rely on a limited set of predefined points to describe a shape, which can be effective for simple shapes but often fails to capture the full geometric complexity of more complex forms. Since only specific points are used, subtle shape variations may be overlooked, leading to a loss of important morphological information, especially in structures including various curved surfaces. Additionally, the accuracy of landmark-based methods depends on the number and placement of landmarks. Too few points can result in missing important details, while increasing the number of landmarks requires more manual effort and can lead to potential errors. In contrast, landmark-free methods utilize dense point clouds (meshes) to analyze the entire shape rather than just discrete points. This allows for a more detailed representation of complex shapes, such as the distal radius. By considering the entire surface, landmark-free approaches provide a more comprehensive analysis of shape variations. By enhancing the accuracy, precision, and efficiency of shape analysis compared to manual, landmark-based approaches, the average degree of interindividual mirror symmetry of distal radii and the distal radius articular surface, as well as the degree of deviation per patient to the mean of a respective population can be specified. Even if the morphometric analysis does not rely on any landmarks, the RS and DSN described by Gray et al. [19] (as well as the centroids for the atlas analysis) were needed in the data preprocessing to achieve a rigid alignment in 3D space. Although the very small amount of landmarks represents a minimal source of error, a fully automated rigid alignment without the use of these landmarks could be advantageous. Such an approach might reduce the need for manual identification and placement of landmarks, speeding up the process and minimizing variability between different users, enhancing reproducibility.

In regard to the interindividual mirror symmetry of the distal radius and its articular surface, our data align with those of previous studies [18,19].

Our findings prove a notable mirrored symmetry within the distal radii of individual patients, especially in the region of the joint surfaces. On average, there is a slight variation of 0.53 ± 0.39 mm between the examined articular surfaces of a patient’s radius. This difference falls well below our defined cut-off of 1 mm. These results suggest that the unaffected contralateral radius can serve as a highly accurate reference for the fractured radius, leading to a favorable postoperative clinical outcome.

When assessing the entire distal radius, we discovered greater point-to-point deviations, in both the pairwise and atlas analyses, in the proximal part, bordering the metaphyseal region. This is probably caused by the alignment of the meshes in 3D space by the anatomical RS and DSN landmarks at the very distal edges of the distal radius joint surface. Consecutively minor discrepancies carry more weight the more proximally they are located relative to the landmarks. This concurs to the results of Hong et al. [18]. Consistent with that hypothesis, our analysis revealed a high level of intraindividual correspondence on the joint surfaces (0.46 ± 0.30 mm). This is consistent with the results presented by Gray et al. [19]. Given that the integrity of the distal radius joint hinges primarily on the shape of its articulating components, the symmetry of the radial joint surface holds particular importance. Our analysis indicates minimal deviations in these critical components, suggesting that the contralateral side demonstrates very high symmetry with only minor deviations below our threshold for expected clinical significance.

The atlas shape analysis highlights substantial variations in radial morphology within the population. Furthermore, the PCA demonstrates that the nature of deformations, irrespective of dimensions, also exhibits gender-specific characteristics, highlighting the multifaceted anatomical variations that can pose challenges for standardized joint replacements. Dividing the cohort by age and gender allows us to capture how bone morphology in the distal radius varies due to aging and anatomical differences between males and females. Age-based groups help identify changes related to bone density and joint structure, mostly due to osteoarthritis over time [30], while gender-based groups account for natural anatomical variations influenced by hormonal and muscular factors [31]. This stratification is crucial for understanding the factors that affect distal radius shape.

This insight carries significant implications and underscores the importance of patient-specific, 3D-printed prostheses in the context of new bio-integrative joint replacements.

Our study clearly demonstrates the intra-individual symmetry of the distal radius joint surface in our study population. When designing a novel wrist prosthesis, the main focus should hence be given to the form and orientation of the joint surface rather than the shaft. With osteochondral technologies even a joint-surface only implant like a crown prosthesis seems plausible.

The results also advocate against a generic “one size fits all” approach since it showed significant interindividual differences in the configuration of the articular surface of the distal radius. The patient-specific approach of 3D-printed prostheses offers a viable alternative that will moreover be even more cost-efficient as technology advances.

We preferred manual segmentation over automated segmentation because, in our case, the radiological software produced inaccurate radius segmentations. This issue may be due to the Partial Volume Effect, where dense tissues like bone transition into less dense tissues such as soft tissue, blurring boundaries. The effect is worsened because the images of the radii were extracted from whole-body CT scans rather than isolated wrist CT scans. This broader focus contributes to reduced accuracy in the automated segmentation, as the radii are not specifically targeted, affecting the quality of segmentation results. The study by Cook et al. demonstrated that manual segmentation of bones with more complex anatomy yields more accurate and reliable results than automated segmentation. Additionally, the study found that manual segmentation can achieve high accuracy and a high level of intrauser and interuser precision [23]. While performing a shape analysis on the distal radius, we encountered specific limitations that could be addressed in future work. The precision of the shape analysis between two shapes heavily depends on their initial accurate alignment. If the shapes are not accurately rigidly aligned, the analysis might highlight differences due to positioning rather than actual shape variations. In our study, this alignment was achieved using anatomical landmarks that were manually placed by a medical expert on the articular surface of the distal radius, as well as the centroid of the shape. However, any minor errors in landmark placement could affect this alignment. Exploring automatic methods for aligning the distal radius shapes could lead to a more robust shape analysis. This alignment could potentially be achieved by training a deep learning network for medical image registration, as described in [32,33]. Additionally, we did not consider factors such as height and weight in the interindividual comparison. These variables could also potentially influence the ultimate bone shape and size, regardless of gender and age.

The relatively small patient population presents a limitation of this study. Thus, the sample size may not capture the full range of variability present in a larger, more diverse cohort. Furthermore, the diversity within our population in terms of occupation, lifestyle, or biomechanical loading patterns was not assessed. These factors could influence the observed mirror symmetry and deformation patterns, as different levels and types of mechanical stress on the wrist may lead to variations in bone geometry and symmetry. Future studies with larger and more diverse populations are needed to evaluate the potential impact of these variables on bone deformation, improving the generalizability of our findings.

## 5. Conclusions

Using a technique of landmark-free morphometrics, this study showed a high symmetry of the distal radius joint surfaces within individuals. These results support using the contralateral side to achieve an anatomically accurate reconstruction of the radius. We hope that future research will lead to clinical studies exploring further applications of these findings in clinical settings and their relevance for 3D-printed biocompatible joint replacements. We recommend further research into the application of these methods to enhance treatment outcomes.

## Figures and Tables

**Table 1:** Parameters for Deformetrica pairwise registration. All parameters used for the pairwise registration that are different from the default parameters set by Deformetrica.

Choice of Hyperparameters for Optimizing Deformetrica Computations					
Template Parameters		Deformation Parameters		Optimization Parameters	
Deformable object type	Surface mesh	Kernel width	2	Optimization type	Gradient ascent
Attachement type	Varifold	Number time points	10	Convergence tolerance	1 × 10^−4^
Kernel width	4	Kernel type	Keops	Gpu mode	Full
Kernel type	Keops			Freeze controle points	On
Noise std	0.0005				

**Table 2:** Pairwise mean Euclidean distance and standard deviation of (a) distal radius and (b) articular surface. Mean distance and standard deviation in mm between corresponding points of the left and right radius and articular surface within one patient and for the four groups of patients.

**(a) Mean Eucidean disatnce (in mm) between left and right distal radius for each patient**							
**Males under 40**		**Males over 40**		**Females under 40**		**Females over 40**	
Patient	Mean (SD)	Patient	Mean (SD)	Patient	Mean (SD)	Patient	Mean (SD)
001	0.58 (0.44)	011	0.41 (0.31)	021	0.49 (0.35)	031	0.62 (0.43)
002	0.76 (0.51)	012	0.78 (0.49)	022	0.55 (0.41)	032	0.51 (0.37)
003	0.64 (0.43)	013	0.55 (0.40)	023	0.31 (0.27)	033	0.55 (0.35)
004	0.75 (0.46)	014	0.76 (0.54)	024	0.42 (0.33)	034	0.65 (0.47)
005	0.51 (0.33)	015	0.76 (0.51)	025	0.58 (0.33)	035	0.49 (0.34)
006	0.37 (0.21)	016	0.44 (0.29)	026	0.52 (0.37)	036	0.54 (0.39)
007	0.44 (0.30)	017	0.48 (0.29)	027	0.41 (0.29)	037	0.40 (0.31)
008	0.68 (0.46)	018	0.60 (0.36)	028	0.30 (0.20)	038	0.33 (0.21)
009	0.49 (0.28)	019	0.37 (0.23)	029	0.49 (0.35)	039	0.65 (0.49)
010	0.43 (0.30)	020	0.59 (0.48)	030	0.44 (0.30)	040	0.41 (0.28)
	0.56 (0.40)		0.57 (0.43)		0.45 (0.34)		0.52 (0.39)
**(b) Mean Euclidean distance (in mm) between left and right distal radius articular surface for each patient**							
**Males under 40**		**Males over 40**		**Females under 40**		**Females over 40**	
Patient	Mean (SD)	Patient	Mean (SD)	Patient	Mean (SD)	Patient	Mean (SD)
001	0.29 (0.21)	011	0.35 (0.22)	021	0.46 (0.30)	031	0.51 (0.27)
002	0.72 (0.39)	012	0.70 (0.32)	022	0.44 (0.31)	032	0.45 (0.27)
003	0.60 (0.33)	013	0.41 (0.22)	023	0.32 (0.21)	033	0.45 (0.24)
004	0.74 (0.36)	014	0.51 (0.34)	024	0.27 (0.17)	034	0.63 (0.37)
005	0.41 (0.25)	015	0.72 (0.32)	025	0.51 (0.26)	035	0.37 (0.21)
006	0.34 (0.19)	016	0.35 (0.20)	026	0.56 (0.32)	036	0.52 (0.33)
007	0.38 (0.21)	017	0.50 (0.23)	027	0.42 (0.29)	037	0.41 (0.28)
008	0.67 (0.33)	018	0.51 (0.25)	028	0.29 (0.19)	038	0.36 (0.20)
009	0.40 (0.23)	019	0.32 (0.21)	029	0.36 (0.21)	039	0.34 (0.25)
010	0.36 (0.21)	020	0.42 (0.27)	030	0.35 (0.23)	040	0.37 (0.20)
	0.50 (0.33)		0.49 (0.30)		0.40 (0.27)		0.44 (0.28)

**Table 3:** Results of Welch’s t-tests conducted on the first and second principal components for each pair of groups. Values that are beneath the significance level of 0.05 are bold.

Group 1	Group 2	PC1
Males under 40	Males over 40	0.309
Males under 40	Females under 40	**0.003**
Males under 40	Females over 40	0.100
Males over 40	Females under 40	**0.002**
Males over 40	Females over 40	**0.023**
Females under 40	Females over 40	**0.010**

**Table 4:** Mean distance and standard deviation in mm between corresponding points of the mean distal radius and distal radius of a patient. Results for distances between (a) the whole distal radii and (b) the articular surface for the left and right sides.

**(a) Mean Eucidean disatnce (in mm) between mean-distal radius and distal radius of each patient**							
**Males under 40**		**Males over 40**		**Females under 40**		**Females over 40**	
Patient	Mean (SD)	Patient	Mean (SD)	Patient	Mean (SD)	Patient	Mean (SD)
001 l	0.47 (0.37)	011 l	2.03 (1.20)	021 l	0.77 (0.45)	031 l	0.89 (0.68)
001 r	0.74 (0.48)	011 r	1.97 (1.14)	021 r	0.73 (0.47)	031 r	0.90 (0.60)
002 l	1.01 (0.66)	012 l	1.21 (0.82)	022 l	0.54 (0.36)	032 l	0.67 (0.50)
002 r	0.55 (0.50)	012 r	0.76 (0.53)	022 r	0.72 (0.55)	032 r	0.71 (0.52)
003 l	1.07 (0.68)	013 l	2.08 (0.98)	023 l	0.98 (0.73)	033 l	0.50 (0.36)
003 r	1.39 (0.75)	013 r	2.08 (0.99)	023 r	1.14 (0.76)	033 r	0.63 (0.43)
004 l	1.40 (0.99)	014 l	0.66 (0.50)	024 l	1.02 (0.63)	034 l	0.73 (0.41)
004 r	1.35 (0.86)	014 r	0.86 (0.53)	024 r	0.93 (0.56)	034 r	0.67 (0.41)
005 l	0.83 (0.65)	015 l	1.63 (0.87)	025 l	1.38 (0.73)	035 l	0.85 (0.48)
005 r	1.00 (0.69)	015 r	1.50 (0.94)	025 r	1.50 (0.91)	035 r	0.83 (0.47)
006 l	0.99 (0.59)	016 l	1.33 (0.69)	026 l	0.55 (0.44)	036 l	1.04 (0.71)
006 r	1.27 (0.67)	016 r	1.54 (0.82)	026 r	0.68 (0.45)	036 r	0.98 (0.78)
007 l	1.02 (0.63)	017 l	1.05 (0.72)	027 l	1.12 (0.62)	037 l	1.03 (0.53)
007 r	1.10 (0.55)	017 r	0.86 (0.50)	027 r	1.03 (0.70)	037 r	0.84 (0.52)
008 l	0.71 (0.43)	018 l	0.39 (0.29)	028 l	0.94 (0.57)	038 l	0.81 (0.59)
008 r	0.56 (0.43)	018 r	0.64 (0.48)	028 r	1.08 (0.59)	038 r	0.92 (0.59)
009 l	0.49 (0.35)	019 l	0.52 (0.35)	029 l	0.66 (0.38)	039 l	0.64 (0.45)
009 r	0.59 (0.39)	019 r	0.49 (0.42)	029 r	0.92 (0.47)	039 r	0.59 (0.47)
010 l	0.69 (0.56)	020 l	1.45 (0.93)	030 l	0.87 (0.50)	040 l	1.56 (0.85)
010 r	0.87 (0.63)	020 r	1.45 (0.98)	030 r	1.11 (0.70)	040 r	1.48 (0.72)
	0.91 (0.68)		1.22 (0.95)		0.93 (0.65)		0.86 (0.63)
**(b) Mean Eucidean disatnce (in mm) between mean-distal radius articular surface and distal radius articular surface of each patient**							
**Males under 40**		**Males over 40**		**Females under 40**		**Females over 40**	
Patient	Mean (SD)	Patient	Mean (SD)	Patient	Mean (SD)	Patient	Mean (SD)
001 l	0.38 (0.24)	011 l	1.37 (0.69)	021 l	0.56 (0.31)	031 l	0.57 (0.41)
001 r	0.43 (0.24)	011 r	1.34 (0.69)	021 r	0.54 (0.31)	031 r	0.52 (0.33)
002 l	0.59 (0.36)	012 l	0.89 (0.61)	022 l	0.34 (0.22)	032 l	0.38 (0.26)
002 r	0.30 (0.18)	012 r	0.49 (0.38)	022 r	0.51 (0.37)	032 r	0.47 (0.30)
003 l	0.83 (0.59)	013 l	1.42 (0.61)	023 l	0.56 (0.30)	033 l	0.32 (0.24)
003 r	0.96 (0.55)	013 r	1.49 (0.53)	023 r	0.69 (0.41)	033 r	0.47 (0.29)
004 l	1.33 (0.95)	014 l	0.45 (0.30)	024 l	0.76 (0.44)	034 l	0.49 (0.29)
004 r	0.95 (0.62)	014 r	0.54 (0.28)	024 r	0.74 (0.41)	034 r	0.45 (0.31)
005 l	0.60 (0.49)	015 l	1.33 (0.74)	025 l	0.68 (0.39)	035 l	0.64 (0.37)
005 r	0.51 (0.35)	015 r	1.08 (0.70)	025 r	0.71 (0.47)	035 r	0.68 (0.37)
006 l	0.66 (0.37)	016 l	0.81 (0.51)	026 l	0.47 (0.34)	036 l	0.53 (0.34)
006 r	0.86 (0.47)	016 r	0.88 (0.54)	026 r	0.61 (0.32)	036 r	0.54 (0.37)
007 l	0.67 (0.32)	017 l	0.65 (0.40)	027 l	0.62 (0.31)	037 l	0.64 (0.46)
007 r	0.77 (0.34)	017 r	0.57 (0.33)	027 r	0.60 (0.30)	037 r	0.50 (0.38)
008 l	0.65 (0.36)	018 l	0.29 (0.20)	028 l	0.55 (0.29)	038 l	0.50 (0.31)
008 r	0.38 (0.25)	018 r	0.33 (0.27)	028 r	0.72 (0.33)	038 r	0.62 (0.31)
009 l	0.38 (0.28)	019 l	0.34 (0.23)	029 l	0.57 (0.28)	039 l	0.40 (0.24)
009 r	0.46 (0.28)	019 r	0.43 (0.37)	029 r	0.65 (0.26)	039 r	0.47 (0.32)
010 l	0.58 (0.40)	020 l	0.71 (0.42)	030 l	0.68 (0.31)	040 l	0.75 (0.49)
010 r	0.63 (0.47)	020 r	0.68 (0.37)	030 r	0.86 (0.43)	040 r	0.76 (0.44)
	0.65 (0.50)		0.81 (0.63)		0.62 (0.36)		0.53 (0.37)

**Table 5:** Results of Welch’s t-tests conducted on the first and second principal components of the momenta vectors describing the pairwise deformation. Values that are beneath the significance level of 0.05 are bold.

Group 1	Group 2	PC1
Males under 40	Males over 40	0.155820
Males under 40	Females under 40	**0.000011**
Males under 40	Females over 40	**0.005161**
Males over 40	Females under 40	**0.000000**
Males over 40	Females over 40	**0.000051**
Females under 40	Females over 40	**0.004091**

## Data Availability

The original contributions presented in the study are included in the article. Further inquiries can be directed to the corresponding author.

## References

[1] Court-Brown C.M., Caesar B. (2006). Epidemiology of adult fractures: A review. Injury.

[2] Knirk J.L., Jupiter J.B. (1986). Intra-Articular fractures of the distal end of the radius in young adults. J. Bone Jt. Surg. Am..

[3] Cayci C., Carlsen B.T. (2014). Osteoarthritis of the wrist. Plast. Reconstr. Surg..

[4] Anderson M.C., Adams B.D. (2005). Total wrist arthroplasty. Hand Clin..

[5] Krukhaug Y., Lie S.A., Havelin L.I., Furnes O., Hove L.M. (2011). Results of 189 wrist replacements. A report from the Norwegian Arthroplasty Register. Acta Orthop..

[6] Ward C.M., Kuhl T., Adams B.D. (2011). Five to ten-year outcomes of the Universal total wrist arthroplasty in patients with rheumatoid arthritis. J. Bone Jt. Surg. Am..

[7] Berber O., Garagnani L., Gidwani S. (2018). Systematic Review of Total Wrist Arthroplasty and Arthrodesis in Wrist Arthritis. J. Wrist Surg..

[8] Nydick J.A., Greenberg S.M., Stone J.D., Williams B., Polikandriotis J.A., Hess A.V. (2012). Clinical outcomes of total wrist arthroplasty. J. Hand Surg. Am..

[9] Boeckstyns M.E.H., Herzberg G. (2024). Complications after total wrist arthroplasty. J. Hand Surg..

[10] Reiser D., Fischer P., Pettersson K., Wretenberg P., Sagerfors M. (2023). Total Wrist Arthroplasty With a New Design, 20 Cases With 8-Year Follow-Up. J. Hand Surg. Am..

[11] Vroemen J.C., Dobbe J.G., Jonges R., Strackee S.D., Streekstra G.J. (2012). Three-Dimensional assessment of bilateral symmetry of the radius and ulna for planning corrective surgeries. J. Hand Surg. Am..

[12] Dobbe J.G., Vroemen J.C., Strackee S.D., Streekstra G.J. (2013). Corrective distal radius osteotomy: Including bilateral differences in 3-D planning. Med. Biol. Eng. Comput..

[13] Baker M.I., Walsh S.P., Schwartz Z., Boyan B.D. (2012). A review of polyvinyl alcohol and its uses in cartilage and orthopedic applications. J. Biomed. Mater. Res. B Appl. Biomater..

[14] Dobbe J.G., Vroemen J.C., Strackee S.D., Streekstra G.J. (2014). Patient-specific distal radius locking plate for fixation and accurate 3D positioning in corrective osteotomy. Strat. Trauma Limb Reconstr..

[15] Trumble T.E., Schmitt S.R., Vedder N.B. (1994). Factors affecting functional outcome of displaced intra-articular distal radius fractures. J. Hand Surg. Am..

[16] Bradway J.K., Amadio P.C., Cooney W.P. (1989). Open reduction and internal fixation of displaced, comminuted intra-articular fractures of the distal end of the radius. J. Bone Jt. Surg. Am..

[17] Fernandez D.L., Geissler W.B. (1991). Treatment of displaced articular fractures of the radius. J. Hand Surg. Am..

[18] Hong E., Kwak D.S., Kim I.B. (2021). Morphological symmetry of the radius and ulna-Can contralateral forearm bones utilize as a reliable template for the opposite side?. PLoS ONE.

[19] Gray R.J., Thom M., Riddle M., Suh N., Burkhart T., Lalone E. (2019). Image-Based Comparison Between the Bilateral Symmetry of the Distal Radii Through Established Measures. J. Hand Surg. Am..

[20] Toussaint N., Redhead Y., Vidal-Garcia M., Lo Vercio L., Liu W., Fisher E.M.C., Hallgrimsson B., Tybulewicz V.L.J., Schnabel J.A., Green J.B.A. (2021). A landmark-free morphometrics pipeline for high-resolution phenotyping: Application to a mouse model of Down syndrome. Development.

[21] Zimmer V.A., Oettlé A., Hoffmann J., Thackeray J.F., Zipfel B., Braga J. (2023). Revisiting mandibular symphyseal shape in juvenile early hominins and modern humans using a deformation-based approach. Folia Primatol..

[22] Buckwalter J.A., Martin J.A. (2006). Osteoarthritis. Adv. Drug Deliv. Rev..

[23] Cook D.J., Gladowski D.A., Acuff H.N., Yeager M.S., Cheng B.C. (2012). Variability of manual lumbar spine segmentation. Int. J. Spine Surg..

[24] Schneider P., Eberly D.H. (2002). Geometric Tools for Computer Graphics.

[25] Wang K., Dai J.S. (2023). The dual Euler-Rodrigues formula in various mathematical forms and their intrinsic relations. Mech. Mach. Theory.

[26] Helbert J., D’Amore M., Aye M., Kerner H. (2022). Machine Learning for Planetary Science.

[27] Durrleman S., Prastawa M., Charon N., Korenberg J.R., Joshi S., Gerig G., Trouvé A. (2014). Morphometry of anatomical shape complexes with dense deformations and sparse parameters. Neuroimage.

[28] Bône A., Louis M., Martin B., Durrleman S. (2018). Deformetrica 4: An Open-Source Software for Statistical Shape Analysis.

[29] Schölkopf B., Smola A., Müller K.-R. (1997). Kernel principal component analysis. Artificial Neural Networks —ICANN’97, Proceedings of the 7th International Conference, Lausanne, Switzerland, 8–10 October 1997.

[30] Martel-Pelletier J., Barr A.J., Cicuttini F.M., Conaghan P.G., Cooper C., Goldring M.B., Goldring S.R., Jones G., Teichtahl A.J., Pelletier J.P. (2016). Osteoarthritis. Nat. Rev. Dis. Prim..

[31] Nieves J.W. (2017). Sex-Differences in Skeletal Growth and Aging. Curr. Osteoporos. Rep..

[32] Haskins G., Kruger U., Yan P. (2020). Deep learning in medical image registration: A survey. Mach. Vision. Appl..

[33] Litjens G., Kooi T., Bejnordi B.E., Setio A.A.A., Ciompi F., Ghafoorian M., van der Laak J., van Ginneken B., Sánchez C.I. (2017). A survey on deep learning in medical image analysis. Med. Image Anal..

